# Commentary: Rethinking fast and slow based on a critique of reaction-time reverse inference

**DOI:** 10.3389/fpsyg.2016.01174

**Published:** 2016-08-05

**Authors:** Gordon Pennycook, Jonathan A. Fugelsang, Derek J. Koehler, Valerie A. Thompson

**Affiliations:** ^1^Department of Psychology, University of WaterlooWaterloo, ON, Canada; ^2^Department of Psychology, University of SaskatchewanSaskatoon, SK, Canada

**Keywords:** dual process theories, response time, reaction time, reasoning, decision making

An increasingly common claim among cognitive psychologists is that the human mind is capable of two fundamentally different types of processes (Evans and Stanovich, 2013): Type 1 processing that is triggered autonomously by a stimulus and Type 2 processing that operates on a deliberate level via working memory and that allows for decoupling or override from default (Type 1) outputs. An article recently published in *Nature Communications* by Krajbich et al. (2015; hereafter, KBHF) focused on the use of response time (RT) differences as evidence for these dual-process theories (hereafter, DPT). KBHF outline how some dual-process theorists argue that if RTs are shorter for some response (“A”) than some other response (“B”), then this supports a DPT wherein Response A is intuitive and Response B is deliberative. Then, using economic games and intertemporal choice as examples, KBHF go on to argue that these RT differences can be better accounted for by sequential sampling models (SSM). Specifically, they argue that RT should increase when participants are presented options that are hard to discriminate (e.g., between two equally preferential choices). Thus, SSMs highlight the importance of conflict between choices as an explanation for RT differences. KBHF conclude by strongly cautioning against the use of RT differences to support DPTs.

In this commentary we will briefly discuss two important questions that arise from KBHF's important contribution: (1) Are all cases in which researchers have used RTs to support DPTs undermined by KBHF's analysis? and (2) Are there any RT differences that are more easily explained under DPT than by SSMs? Our overarching goal is to illustrate that the scope of KBHF's analysis is far more limited than would be reasonable surmised by their original article.

## Are all cases in which researchers have used RTs to support DPTs undermined by KBHF's analysis?

As mentioned, KBHF focus on DPTs in which responses are labeled as either “intuitive” or “deliberative.” KBHF accurately demonstrate the problematic nature of inferring that a given response is intuitive or deliberative based on fast or slow RTs (respectively). Indeed, in dual-process research, this practice is known as the labeling fallacy and most commonly occurs in the domain of normative accuracy (with logical responses assumed to be the result of Type 2 processing; e.g., Gibbard, 1990; Epstein, 1994). Crucially, however, this practice has been decried by dual-process theorists (e.g., Evans and Stanovich, 2013), who point out that Type 1 processing can lead to correct responses and Type 2 processing can support biased processing (Evans, 2007; Stanovich, 2011). Unfortunately, KBHF do not mention that there are many DPTs that do not attempt to make such claims. For example, Evans and Stanovich (2013) have claimed that speed and accuracy are correlated but not central factors in what determines the distinction between Type 1 and Type 2 processes. This is an important addendum to KBHF because, based on their analysis alone, it is unclear whether there is good evidence to contend that RT differences should not be used to support *any form of* DPT. They fail to mention that there are any other classes of DPT outside of the limited class that they discuss.

## Are there any RT differences that are more easily explained under DPT than by SSMs?

That KBHF only discussed one class of DPT does not mean that other classes of DPT are necessarily immune from their criticism. Thus, we will highlight a set of empirical results that are *as easily* or perhaps even *more easily* explained under DPTs than by simple SSMs. Our goal is to illustrate not simply that the DPT that KBHF outlined is not representative of the broader literature, but that there is good reason to believe that RTs can be a useful tool in DPT research.

Recent DPTs have focused on the conflict between evidence (or, put differently, response outputs) as a mechanism that *causes* deliberation and, as a result, RT differences (De Neys, 2012, 2014; Sloman, 2014; Handley and Trippas, 2015; Pennycook et al., 2015; for an important predecessor, see Sloman, 1996; for an example in the realm of cooperation, see Evans et al., 2015)^1^. Thus, like SSMs, there are DPTs that focus on response competition (manipulated experimentally) and not on labeling responses as intuitive or deliberative. Consider, for example, research on base-rate problems. These problems describe a conflict between the probability of group membership based on a base-rate (e.g., 99.5% chance that Paul is a nurse) and a set of stereotypes that are consistent with an alternative (e.g., Paul seems more like a doctor). The typical finding for problems of this type is that people have a strong preference for the stereotypical information (e.g., 81% stereotypical responses in De Neys and Glumicic, 2008, Experiment 1). Nonetheless, for example, De Neys and Glumicic found that participants took (roughly) 5 additional seconds to give stereotypical responses if they were presented conflicting base-rate information (relative to non-conflict versions of the problems). How can this RT difference be explained?

Based on KBHF's characterization of DPT, the conclusion from DPT proponents would be that stereotypes are “Type 1” responses whereas base-rates are “Type 2” responses. This, however, is not the case. De Neys and Glumicic's (2008) primary comparison (with respect to RTs) was *not* between base-rate and stereotypical responses, but between incongruent (conflict) and congruent (no-conflict) problems. Specifically, they found that participants take longer when solving problems that contain a conflict between base-rate and stereotypes *regardless of whether they give base-rate or stereotypical responses*. This finding, first reported using RTs, was then conceptually replicated using a wide variety of different tasks (e.g., syllogisms; conditionals; the conjunction fallacy; and ratio bias) and additional measures (e.g., memory recall; verbal protocols; skin conductance response; confidence; fMRI; and ERP; see De Neys, 2012, 2014 for reviews). This supports the contention that the detection of conflict between initial response outputs *causes* Type 2 processing (Pennycook et al., 2015).

We should note that the increase in RT as a function of the conflict manipulation can be accommodated under SSMs. Indeed, KBHF's key demonstration involved a manipulation of choice conflict. Both SSMs and DPTs predict that RT should increase when two choices come into conflict. However, in our view, understanding this conflict at the cognitive level implicates dual-processing. For example Pennycook et al. (2015) argued for a three-stage dual-process model in which conflicting initial choice outputs (Stage 1) may be recognized as such (Stage 2), which then initiates analytic processing (Stage 3). This goes beyond the simple observation that choice conflict causes increased RT.

Importantly, not all patterns of data are equally easy to accommodate under DPTs and SSMs (but see Alos-Ferrer, 2016 for a dual-process drift diffusion model). For example, although De Neys and Glumicic (2008) found evidence for an increase in RT for stereotypical responses to conflict vs. no-conflict problems with extreme base-rates (e.g., 995 lawyers, 5 engineers), this effect diminishes if base-rates are made moderate (e.g., 700 lawyers, 300 engineers; Pennycook et al., 2012, 2015). In theory, this could be accommodated by a SSM wherein participants experience more conflict between stereotypical choices and *extreme* base-rates than they do with *moderate* base-rates. Crucially, however, manipulating the extremity of the base-rates does *not* have an effect on RTs for *base-rate* responses to conflict problems (i.e., the base-rate extremity effect is only evident for stereotypical responses; Pennycook et al., 2012, 2015).

According to Pennycook et al. (2015), this pattern of results occurs because RT for base-rate responses is primarily determined by cognitive decoupling—that is, the time it takes to override the salient stereotype via Type 2 processing (the stereotypes are identical across moderate and extreme base-rate experiments). The stereotypical responses are fundamentally different because at least some of the people who do not spend much time giving stereotypical answers genuinely do not distinguish between conflict and non-conflict problems (i.e., they are not detecting the conflict between their stereotypical answer and presented base-rates). Pennycook et al. (2015) argued that the probability of this happening increases when base-rates are made moderate relative to extreme, hence the aforementioned pattern of results. Thus, RTs can be used in an experimental context to distinguish between conflict detection and cognitive decoupling in DPTs. At the very least, evidence of RT differences as a consequence of response conflict is not necessarily evidence for SSMs and against DPTs.

## Conclusion

In our opinion, there are good reasons to continue using RTs in DPT research. We have focused on one example, but there are other types of evidence to support this contention. For example, Thompson and colleagues (Thompson et al., 2011, 2013; Thompson and Johnson, 2014) found a negative correlation between the “feeling of rightness” associated with an initial response (e.g., stereotypical responses for base-rate problems) and the amount of time spent reasoning when giving a final answer. This finding indicates that metacognitive processes are an important determinant of how much Type 2 processing will be engaged for any given sort of problem. In a different realm altogether, there is evidence that religious believers are less analytic (see Pennycook et al., 2016) and, as a consequence, spend less time when presented with reasoning problems (Pennycook et al., 2013, 2014). These findings were predicted by DPTs, which indicates that the framework has been useful for generating hypotheses to be tested using RTs.

Dual-process theory has proven to be quite popular among psychologists. Naturally, however, not all applications of DPT are created equal. Dual-process research that relies on experimental manipulations and that is not focused on making claims about whether responses are purely “intuitive” or “analytic” are (at least) on the same footing as other decision making models. Further, empirical work that teases apart the cognitive mechanisms that explain why decisions slow in the face of conflicting evidence is required, but in our view DPTs are in a strong position to facilitate this research.

## Author contributions

GP wrote the article. VT, DK, and JF provided critical revisions.

## Funding

The primary author was supported by a Canada Graduate Scholarship from the Natural Sciences and Engineering Research Council of Canada.

### Conflict of interest statement

The authors declare that the research was conducted in the absence of any commercial or financial relationships that could be construed as a potential conflict of interest.

## Footnotes

^1^The underlying claim is that an increase in RT indicates a potential increase in deliberative Type 2 processing. Notably, this is not the same as arguing that longer RTs automatically indicate the presence of Type 2 processes (and implied absence of Type 1 processing). That is, a relative increase in Type 2 processing should be reflected in longer RTs, but this does not mean that the associated response was not initially generated autonomously and intuitively.
